# A New Antipruritic Remedy

**Published:** 1875-10

**Authors:** 


A NEW ANTIPRURITIC REMEDY. By L. Duncan Bulkley, A.M., M.D. New York. Pamphlet; pp. 4.

